# 

**Published:** 1887-07

**Authors:** 


					﻿olume LVJ
JULY, 1887.
[Number 1.
ham V	O
MEDICAL ;
' Journal
EXAMINER
EDITOR:
S. J. JONES, M.D., LL.D.
j i	1 ■ * ■ min i • * •»"* * H 1 • H i »im i iiit i > i>'‘	t >\Hi / it* ‘ t i ti •» * > * * / > iu/t
.^B^3 (LARK g> LONGLEY \O.,(HI(AGO,
I i 111 III i 1111 >11 i 111 ■< . PVBL<ISHE1R'S11hij .....if.,..	|
				

